# Anti-diabetic prospects of dietary bio-actives of millets and the significance of the gut microbiota: A case of finger millet

**DOI:** 10.3389/fnut.2022.1056445

**Published:** 2022-12-22

**Authors:** Vineet Singh, GyuDae Lee, HyunWoo Son, Sliti Amani, Mamta Baunthiyal, Jae-Ho Shin

**Affiliations:** ^1^Department of Applied Biosciences, Kyungpook National University, Daegu, Republic of Korea; ^2^Department of Biotechnology, Govind Ballabh Pant Institute of Engineering and Technology, Ghurdauri, India

**Keywords:** bio accessibility, gut microbes, obesity, diabetes, insulin resistance, inflammation, endotoxemia, finger millet

## Abstract

Finger millet (*Eleusine coracana*) is a staple food in several parts of the world because of its high nutritional value. In addition to its high nutrient content, finger millet contains numerous bioactive compounds, including polyphenol (10.2 mg/g TAE), flavonoid (5.54 mg/g CE), phytic acid (0.48%), and dietary fiber (15–20%). Polyphenols are known for their anti-oxidant and anti-diabetic role. Phytic acid, previously considered an anti-nutritive substance, is now regarded as a nutraceutical as it reduces carbohydrate digestibility and thus controls post-prandial glucose levels and obesity. Thus, finger millet is an attractive diet for patients with diabetes. Recent findings have revealed that the anti-oxidant activity and bio-accessibility of finger millet polyphenols increased significantly (*P* < 0.05) in the colon, confirming the role of the gut microbiota. The prebiotic content of finger millet was also utilized by the gut microbiota, such as *Faecalibacterium*, *Eubacterium*, and *Roseburia*, to generate colonic short-chain fatty acids (SCFAs), and probiotic *Bifidobacterium* and *Lactobacillus*, which are known to be anti-diabetic in nature. Notably, finger millet-induced mucus-degrading *Akkermansia muciniphila* can also help in alleviate diabetes by releasing propionate and Amuc_1100 protein. Various millet bio-actives effectively controlled pathogenic gut microbiota, such as *Shigella* and *Clostridium histolyticum*, to lower gut inflammation and, thus, the risk of diabetes in the host. In the current review, we have meticulously examined the role of gut microbiota in the bio-accessibility of millet compounds and their impact on diabetes.

## 1 Introduction

Diabetes, one of the most common metabolic diseases, results in hyperglycemic conditions caused by insufficient insulin secretion and reduced insulin sensitivity. Diabetes can be divided into two types: type 1 and type 2. Type 1 diabetes is characterized by little or no insulin production owing to complete or partial autoimmune destruction of the insulin-producing pancreatic β-cells (1). However, Type 2 diabetes is more complex, with either insulin resistance developing or reduced sensitivity toward insulin levels, thus resulting in high glycemic conditions (2). Diabetes is a chronic metabolic disease associated with numerous complications, including obesity and lipid metabolism dysfunction, leading to higher triglyceride levels, cardiovascular diseases, and neuropathy owing to higher glucose levels (3, 4). According to WHO data, approximately 422 million people have diabetes, which is responsible for approximately 1.5 million deaths yearly (5).^1^

The etiology of diabetes includes a higher glycemic index, low-grade systemic inflammation, obesity, and higher oxidative stress, which further exacerbates the severity of diabetes. Various dietary strategies (probiotic, prebiotic, and synbiotic) (6, 7) and dietary substances rich in polyphenols efficiently controlled some of the adverse conditions of diabetes (8). Finger millet (*Eleusine coracana*) is a naturally gluten-free cereal grain rich in nutrients. It contains carbohydrates (65–75%), proteins (5–8%), dietary fiber (15–20%), minerals (2.5–3.5%), and Vitamin B complex (9, 10). Among all the cereals, finger millet contains the highest calcium content (337.53–353.41 mg/100 g), together with high phosphorus (314.46–341.70 mg/100 g), zinc (1.96–1.93 mg/100 g), and iron (3.28–3.86 mg/100 g) contents (11). According to the national health service-UK, the daily requirement of above mentioned micro nutrients are 700 mg/Day (Ca), 550 mg/day (phosphorus), 7–9.5 mg/day (Zn), and 8.7–14.8 mg/day (Fe) (12).^2^ Furthermore, finger millet is balanced in its protein content, as it is high in essential amino acids such as methionine, lysine, threonine, and valine, compared with that in other millets (13). Finger millet is also rich in dietary polyphenols (10.2 mg/g GAE), flavonoids (5.54 mg/g CE) (14), and tannins (0.04–3.47%) (15). Bioactive compounds of finger millet are anti-diabetic owing to their high fiber, flavonoid, and polyphenol contents, control hyperglycemic levels, and reduce oxidative stress by acting as an anti-oxidant. Finger millet grains are also rich in phytic acid (0.48%) and trypsin inhibitory factors, adversely affecting the availability of carbohydrates and proteins in the intestine, thereby lowering the glycemic level (9). Gut microbiota increased the catabolism of dietary polyphenols in the gut, and various simple bioactive derivatives were produced, improving bioavailability (16). Additionally, the gut microbiota utilizes dietary fibers to produce short-chain fatty acids (SCFAs; acetic acid, propionic acid, and butyric acid) that modulate insulin sensitivity, low-grade systemic inflammation, and glucose-lipid metabolism (17). Moreover, studies confirmed the synergistic roles of butyric acid and phenolic acids (benzoic acid, phenylacetic acid, and phenylpropionic acid) resulted in strong anti-inflammatory effects (18).

**FIGURE 1:**
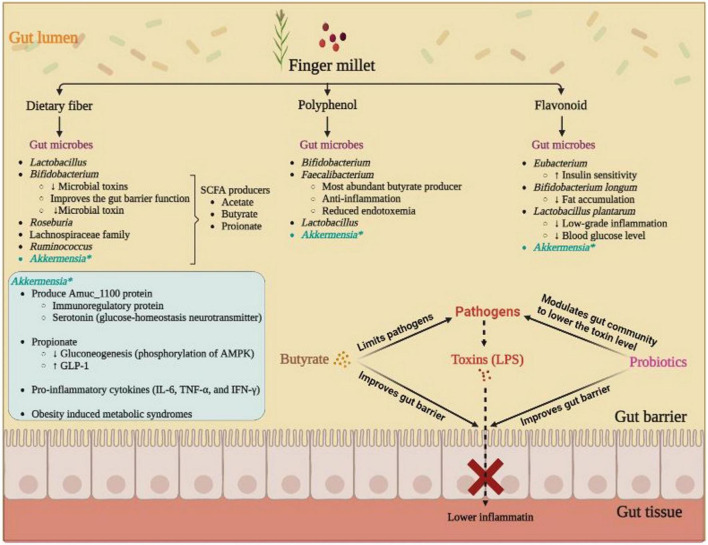
Role of finger millet bio-active molecule induced gut microbes in alleviation of diabetes; butyrate producers *Faecalibacterium*, *Roseburia*, and *Eubacterium* play a crucial role in reducing systemic inflammation by maintaining the gut barrier function to avoid the gut invasion by pathogenic microbes and toxins. Mucus degrading *Akkermansia*, participates in glucose-homeostasis by means of Amuc_1100 protein and regulates the blood glucose and lowers the gut inflammatory load by releasing propionate. Probiotics (*Bifidobacterium* and *Lactobacillus*) also improves the toxin level by modulating the gut community, i.e., by lowering the various possible opportunistic pathogen, and simultaneously enhancing the beneficial microbes.

Finger millet, a rich source of phenolic acids and prebiotic dietary fibers, can be readily utilized by the gut microbiota to generate SCFAs, specifically butyrate (19). Numerous other finger millet bio-actives also modulated the gut communities by enhancing certain gut microbes and inhibiting others, possibly inducing an anti-diabetic effect (20). Therefore, the present review sought to understand the health benefits of finger millets and their role in gut microbiota, specifically against diabetes (Figure 1).

## 2 Bioactive compounds in finger millet and their role in diabetes

Finger millet is a staple diet in various countries, including India, Nepal, Sri Lanka, and parts of Africa. However, consumption of finger millet is increasing in other parts of the world, including the Middle East and Europe, owing to public health awareness. In addition to their high nutritional content, finger millet grains are rich in dietary bioactive compounds such as polyphenols and dietary fibers (21). These molecules are further metabolized in the colon by the gut microbiota into bioactive molecules such as SCFAs and small-chain phenolic acids (21).

### 2.1 Dietary fiber

Dietary fibers are indigestible oligosaccharides or polysaccharides that cannot be digested by the human digestive system and act as roughage maintaining stool movement and cleaning the digestive tract. By nature, dietary fibers can also be prebiotic if they are metabolized by the gut microbiota, such as *Faecalibacterium*, *Roseburia*, *Anaerostipes*, and *Ruminococcus*, members of the family *Lachnospiraceae*, and members of *the Clostridium* IV cluster, in the colon to produce different SCFA including acetic acid, propionic acid, and butyric acid (22). In colonic fermentation, gut-microbiota hydrolyzes non-digestible dietary fiber into constituent monosaccharides through an exclusive set of enzymes, that are not present in humans. These enzymes belong to the carbohydrate-active enzyme (CAZyme), which hydrolyzes the complex fiber into respective monosaccharides (23). These hydrolyzed monosaccharides were further utilized by the microbes to generate different SCFAs *via* different pathways (Figure 1). For instance, gut microbes can produce acetate from the pyruvate *via* acetyl-CoA, propionic acid from succinate pathway, acrylate pathway, and propanediol pathway (24). While butyric acid is produced by condensing the two molecules of acetyl-CoA with simultaneous reduction to butyryl-CoA, which will be converted into butyric acid by microbial phosphotransbutyrylase, and butyrate kinase (24). Normally, the gut SCFAs (acetic acid, propionic acid, and butyric acid) are in a molar ratio of 60:20:20 (25). SCFA’s also reduces gut permeability to lower the toxin flow-through from the gut lumen to colonic tissues and liver (26), as they enhance the tight junction protein such as claudin-2, claudin-7, zonula occludens-1 (ZO-1), and occludin (27, 28).

In the gut, only a small fraction of SCFAs is metabolized by the microbes, which distinctly affects the gut microbiome, and majority of SCFAs are passively diffused to colonocyte cells *via* monocarboxylate transporter. Colonocyte cells are the intestinal enterocyte which utilizes SCFAs, specifically butyric acid to generate energy and stabilizes hypoxia-inducible factor (HIF) to maintain the anaerobic condition in gut, and the remaining SCFAs are transported to the portal system. In the liver also, butyric acid is utilized by the hepatocyte cells to produce energy, and the remaining SCFAs were transported to peripheral tissues. Other than that, SCFAs also act as signaling molecules and activate G-protein coupled receptors (GPR41 and GPR43) in colonocytes and adipocytes. Upon activation, GPR41 and GPR43 stimulate the release of glucagon-like peptide-1 (GLP-1) and peptide yy (PYY) hormones from the colonocyte, which induces satiety, stimulates insulin secretion, and decreases glucose level by decreasing glucagon secretion from the pancreas which generates the glucose from glycogen, to alleviate diabetes. In adipocytes, SCFA activated GPR41 and GPR43 induce higher levels of leptin hormone that also regulates glucose-energy homeostasis by increasing triglyceride hydrolysis and oxidation of free fatty acids in adipose tissue, thus promoting adipogenesis and inhibiting low-grade inflammation (29, 30). In addition, leptin also inhibits hunger to control the excess food intake and thus postprandial blood sugar level (31).

Other than that, SCFA-activated GPRs (GPR41, GPR43, and GPR109) also limit colonic inflammation, as butyric acid inhibits lipopolysaccharide (LPS) induced NF-kB activation through GPR109 (32). Additionally, butyric acid downregulates the LPS-induced pro-inflammatory cytokines such as IL-6, IL-12 (33), and IL-8 by influencing the macrophages and neutrophils, respectively (34). Moreover, butyric acid also helps in mucosal healing by stimulating the migration of epithelial cells (35). Thus, SCFAs maintain gut homeostasis by alleviating gut inflammation, limiting the prevalence of pathogenic microbes, and controlling gut permeability. They also participate in metabolic reactions, particularly butyric acid, which is utilized by the gut colonocyte cells to render the gut environment strictly anaerobic and thus limit the abundance and growth of possible pathogenic microbes such as *Escherichia coli* and *Salmonella* (36). As mentioned before, SCFAs can also regulate the host-energy metabolism and thus diet-induced obesity (30), which is a major risk factor for diabetes (37). Previous studies have demonstrated that SCFAs are also effective in alleviating chronic inflammation.

In finger millet, the main constituent of the carbohydrates is starch (65–75%), and the non-starchy polysaccharides, arabinoxylan, and β-glucan constitute the dietary fiber (15–20%) (38). According to their solubility in water, dietary fiber can be divided into soluble and insoluble categories. The soluble and insoluble dietary fiber fractions vary in finger millet. For example, Chethan and Malleshi (39) reported that the dietary fiber in finger millet was 15.7% insoluble and 1.4% soluble, whereas Shobana and Malleshi (40) reported 19.7% insoluble and 2.5% soluble dietary fiber in finger millet. The formation of resistant starch, another form of soluble fiber, which is a product of partial/least hydrolysis of starch during gastric digestion (41), also contributed to the overall dietary fiber content of finger millet (40). Soluble dietary fiber exhibited gel-forming properties by absorbing water and thus increasing food-viscosity that inhibited macronutrient absorption, reduced dyslipidemia, reduced the postprandial glucose response, and could be metabolized by the gut microflora (*Bacteroides* and *Bifidobacterium*) during colonic fermentation to generate SCFAs (42). Soluble dietary fibers decrease the absorption of bile acid in the small intestine (ileum) and increase the loss of bile acid, resulting in increased hepatic bile synthesis using intracellular cholesterol, which lowers the serum cholesterol level (43). However, insoluble dietary fibers, including cellulose and hemicellulose, were more prone to colonic fermentation by the gut microbiota and affects insulin sensitivity positively (44). In human gut, cellulose and hemicellulose are specifically metabolized by the member of the genus *Ruminococcus*, *Prevotella*, and *Clostridium* (45, 46). Insoluble fibers also aid in removing toxins from the intestinal tract through several mechanisms (47). They increase intestinal transit and enhance fecal bulk by increasing its water-holding capacity (48). However, prebiotic-resistant starch, that escapes enzymatic digestion, can be utilized by the gut microbiota to enhance the proportion of beneficial microbes such as probiotic *Bifidobacterium*, *Lactobacillus*, and *Akkermansia* (49). Studies have reported that millet-based preparations with a high dietary fiber content lower the glycemic responses and are therefore recommended for diabetic individuals (50). Several mechanisms have been proposed to promote the beneficial metabolic effects of finger millet dietary fiber, including altered lipid metabolism, altered bile acid metabolism, and improvement in glucose levels (9). In addition, being high in microbiota-accessible dietary fiber, the gut microbiota is considered to be involved in the anti-diabetic and anti-obesity effects of finger millet.

### 2.2 Polyphenols

Only 5–10% of polyphenols are absorbed in the small intestine, with the remaining polyphenols (approximately 90%) reaching the large intestine (51). In the colon, accumulated polyphenols are actively metabolized by the gut microbiota (*Akkermansia, Lactobacillus* sp., and *Eubacterium* sp.) into smaller phenolic metabolites such as urolithins from ellagic acid (52), and protocatechuic acid from quercetin (53), which has health-benefiting effects. The gut microbes hydrolyze the polyphenols using (poly)phenol-associated enzymes (PAZymes) (54), cleaving glycosidic bonds and breaking complex heterocyclic compounds into smaller ones that are more active than the parent compound (55). In addition to the smaller phenolic compounds, polyphenols are prebiotic, and produce SCFA upon metabolization by the gut microbiota such as *Faecalibacterium*, *Eubacterium*, *Roseburia*, and members of the family Ruminococcaceae (51, 56). Studies have confirmed that different polyphenols such as catechin, ellagic acid, and ferulic acid affected the Bacteroides/Firmicutes ratio and altered the gut communities differently (57). For example, catechin (150 mg/L) significantly inhibited (*P* < 0.05) the pathogenic *Clostridium histolyticum*, while encouraging the growth of the probiotic, *Bifidobacterium*, and the microbes of the butyrate-producing bacteria, *Clostridium coccoides-Eubacterium rectale* group (58). Further, upon diffusion through colonic epithelial, they reach the liver *via* the portal system and can downregulate the liver genes associated with obesity and lipid metabolism (51). In particular, expression of sterol regulatory element binding protein 1 (SREBP1), fatty acid synthase (FAS), and stearyl CoA desaturase 1 is downregulated in the liver due to dietary polyphenols (55). In addition, ferulic acid; an abundant polyphenol in millets, also improves glucose and lipid homeostasis by inhibiting expression of the protein associated with gluconeogenesis such as phosphoenolpyruvate carboxylase (PEPCK) and glucose-6-phosphatase (G6P), to lower blood glucose (59).

Finger millet is a rich source of polyphenol (3.05–10.20 mg/g GAE; GAE, gallic acid equivalent) (14, 60) and flavonoids (2–5.54 mg/g CE; CE, catechin equivalent) (14, 61). However, different polyphenol levels have been reported in different finger millet varieties (15). Even in finger millet grain, the distribution of polyphenols was not uniform and was reported to be the highest in the seed coat, which also varied among different varieties (0.3 ± 0.1–2.3 ± 0.2% g) (39). The polyphenols present in finger millet are listed in Table 1. In finger millet grain, phenolic compounds are present in free, soluble-conjugated, and insoluble forms. Among these, ferulic acid, caffeic acid, and p-coumaric acid were the major insoluble bound polyphenols, whereas protocatechuic acid, gallic acid, and caffeic acid were the major free polyphenols of finger millet (62). Polyphenols (250 mg/day in diet) are crucial in controlling diabetes as they effectively reduce insulin resistance (63, 64) and regulate glucose and lipid metabolism (65). Similarly, finger millet polyphenols helped maintain the lipid metabolism as ferulic acid, a major polyphenol in finger millet (35.19–67.36 mg/100 g of dry weight), decreased the total cholesterol level (9). Gallic acid exhibited various biological properties, such as antioxidant (50–200 μM) (66), anti-inflammatory (25–100 μM) (67), and anti-microbial properties (2–8 mg/ml) (68). Catechin-rich (582.8 mg/340 ml) beverages reportedly improve obesity and blood glucose levels (69). This was achieved by enhancing the expression of adiponectin, the anti-diabetic protein, which increases insulin sensitivity and adipocyte differentiation, thereby lowering plasma glucose and free fatty acid levels (70). In an induced high-fat diet murine study, the anti-obesity effect of ferulic acid (50 mg/kg of body weight) was also confirmed (71). Finger millet polyphenols can also be metabolized by gut microbes (72) and could thus participate in the anti-diabetic effects of finger millet, as will be discussed later.

**TABLE 1:** Various polyphenolic compounds present in finger millet extract (n-hexane) (123).

Class	Compound	Structure	Chemical formula	Concentration (ppm)
Hydroxybenzoic acid	Gallic acid	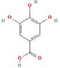	C_7_H_6_O_5_	3.57 ± 0.5
	Vanillic acid	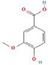	C_8_H_8_O_4_	32.01 ± 1.76
	Protocatechuic acid	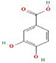	C_7_H_6_O_4_	343.4 ± 7.4
Flavanols	Catechin	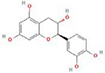	C_15_H_14_O_6_	5281.7 ± 223.7
	Epicatechin	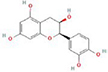	C_15_H_14_O_6_	5.95 ± 0.05
	Quercetin	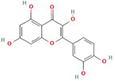	C_15_H_10_O_7_	12.76 ± 1.96
	Homovanillic acid	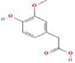	C_9_H_10_O_4_	29.09 ± 8.41
	Naringin	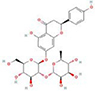	C_27_H_32_O_14_	218.57 ± 2.96
	Taxifolin	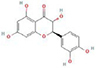	C_15_H_12_O_7_	12.22 ± 0.02
Hydroxycinnamic acid	Ferulic acid	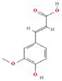	C_10_H_10_O_4_	17.95 ± 2.45
	p-Coumaric acid	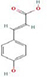	C_9_H_8_O_3_	2 ± 0.004
Benzoic acids ester	Methyl vanillate	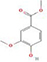	C_9_H_10_O_4_	2.51 ± 1.01
	Chlorogenic acid	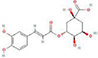	C_16_H_18_O_9_	134.2 ± 1.46

## 3 Bio digestibility and bio-accessibility of finger millet bio-active compounds

The portion of dietary fiber and polyphenols released after gastrointestinal digestion (Table 2) or by the action of the gut microbiome is bio-accessible and metabolically bioactive (14). Generally, the gastrointestinal release of bioactive molecules, such as phenolic acid, from digested finger millet is low owing to the formation of complexes with the dietary fiber (arabinoxylan) (9). Furthermore, the presence of anti-nutritional factors in finger millet, such as phytic acid and tannin, hinders the availability of nutrients and decreases nutritional availability and the digestibility of starch and protein (73). In the body, enzymatic digestion of easily digestible starch and protein results in a sudden increase in blood glucose, however, owing to the presence of anti-nutritional factors, finger millet flour has a lower glycemic index (42.24) than wheat flour (62.59), proso millet flour (52.49), foxtail millet flour (49.28), and barnyard millet flour (47.95) (74), thereby indicating its nutraceutical potential (75). Although finger millet contains 22 polyphenols, only 13 are bio-accessible (14). The bio-accessible content of finger millet polyphenol and flavonoid is around 2.65 and 1.09 mg/g, respectively, which does not change with roasting (14). However, gut microbiota can enhance the availability of high molecular weight polyphenolic compounds by degrading them into simpler forms (72). In addition, studies reported that polyphenolic and antioxidant levels of finger millet increase significantly (*P* < 0.05) after digestion and increases after fermentation by gut microbes in the colon (76). Another study confirmed a significant increase (*P* < 0.05) in polyphenolic levels and anti-oxidant activity of finger millet flour upon fermentation (38). Colonic fermentation of finger millet polyphenols may favor certain microbes with antidiabetic properties to lower the risk of diabetes.

**TABLE 2:** Effect of various processing methods on bio-accessible content of finger millet polyphenols and flavonoids.

Processing method	Polyphenol (mg/g GAE)	Bio-accessible polyphenol (mg/g GAE)	Bio-accessibility (%)	Flavonoid (mg/g CE)	Bio-accessible flavonoid (mg/g CE)	Bio-accessibility (%)
Native	10.2 ± 0.21	2.65 ± 0.21	25.5	5.54 ± 0.40	1.09 ± 0.08	19.7
Sprouting/germination	6.78 ± 1.04*	2.49 ± 0.11	36.8	3.33 ± 0.32*	0.81 ± 0.14*	24.3
Roasting	11.9 ± 0.67*	2.46 ± 0.16	20.7	4.92 ± 0.19	1.14 ± 0.11	23.2
Pressure cooking	5.95 ± 0.65*	1.84 ± 0.12*	30.9	1.78 ± 0.19*	0.89 ± 0.13*	50.3

GAE, gallic acid equivalent; CE, catechin equivalence. *Significantly different (*P* < 0.05) from native (14).

## 4 Anti-diabetic role of finger millet-induced gut microbiota

Microbial-dysbiosis in the gut of patients with diabetes and the role of the gut microbiota in regulating various diabetes-associated metabolisms, such as glucose homeostasis, oxidative stress, and T2D-associated pro-inflammatory cytokines (IL-1 and IL-6), is well documented (2). Studies have demonstrated that gut dysbiosis in diabetic patients can be alleviated by certain diets or dietary interventions such as prebiotics (77), polyphenols (78), flavonoids (79), and glucosinolates (80, 81). Fecal microbiome transplantation (FMT) therapy also effectively controls the etiology of diabetes, such as insulin resistance and inflammation, providing evidence of the significance of the gut microbiota in diabetes (82, 83). As discussed previously, finger millet is explicitly enriched in microbiota-accessible compounds (prebiotics and polyphenols) that are metabolized by the gut microbiota and benefit the health of the host. Prebiotics and polyphenols reportedly ameliorate diabetes and its associated co-morbidities, such as obesity, low-grade inflammation, and blood glucose level regulation (2). Recent studies on finger millet have demonstrated its importance in diabetes and its ability to modulate the gut microbial community (Table 3). Along with dietary fibers and polyphenols, finger millet is also rich in antioxidants and metabolically active compounds (vitamins E, A, and B), which also play a significant role in lowering oxidative stress and maintaining a healthy gut microbiome (84, 85).

**TABLE 3:** Studies showing anti-diabetic significance of finger millet along with its effect on gut microbiota.

Study	Model/design	Anti-diabetic effect	Impact on gut microbiota	References
Finger millet bran treatment for high-fat diet-fed mice	• HFD mice model with negative control 12-week duration	• Improved lipid profile and anti-inflammatory status • Lowered oxidative stress • Prevented weight gain	• ↑ Abundance of *Lactobacillus*, *Bifidobacterium*, and *Roseburia* • ↓ Abundance of *Enterobacter*	(19)
Effect of Finger millet polyphenols on high-fat diet induced metabolic activities	• Polyphenol-rich extracts made using n-hexane • HFD mice model • 2-week duration	• Prevented fat deposition, and adipocyte hypertrophy • Decrease in systemic inflammation • Improved body mass index	• ↑ Bacteroidetes/Firmicutes ratio	(123)
Effect of finger millet arabinoxylan on HFD fed mice	• Oral gavage of 0.5 and 1.0 g/kg bodyweight • 10-week duration	• Improved glucose and serum lipid profile • Improved liver and white adipose tissue gene expressions • Decrease in hepatic lipid accumulation and inflammation	• ↑ Firmicutes ↑ Lachnospiraceae family • ↑ Microbial richness and diversity	(20)
Prebiotic activity of purified finger millet xylobiose	• Enzymatic digestion and *in vitro* fermentation • Enzymatic hydrolysis of purified xylanase (240 μg) at 50°C for 150 min	• SCFA generation; specially acetate	• ↑ *Bifidobacterium* and *Lactobacillus*	(124)
Role of finger millet β-glucan	• *In vitro* model • α-Amylase and α-glucosidase inhibition at 1.23 and 1.42 μg/ml, respectively • Antibacterial activity at <70 μg/ml	• Inhibits the activity of α-amylase and α-glucosidase • Antidiabetic and antioxidant activity	• ↓ *Lysinibacillus fusiformis* and *Enterococcus faecalis* • ↓ *Proteus vulgaris* and *Shigella sonnei*	(89)
Synbiotic effect of Finger millet porridge	• *In vitro* model • Presence of probiotic	• Antioxidant activity	• ↓ *Shigella flexneri*, *Staphylococcus aureus*, *Salmonella typhi*, and *Escherichia coli*.	(125)
Synbiotic effect of fermented finger millet milk	• *In vitro* model • Toned milk with 20% finger millet flour, along with *Streptococcus thermophilus* MTCC 5460 and *Lactobacillus helveticus* MTCC 5463	• Antioxidant, antimicrobial, and antidiabetic potential • Angiotensin-converting enzyme (ACE) inhibitory	• *Staphylococcus aureus*, *Salmonella typhi*, and *E. coli*	(126)

### 4.1 Significance of finger millet-induced gut microbiota to control obesity and blood glucose levels

Obesity develops as a result of excessive fat accumulation in the body and is directly associated with various metabolic disorders, including diabetes and associated comorbidities. Obesity or excessive fat deposition results in dyslipidemia, a disturbed lipid metabolism inducing excessive triglycerides and free fatty acids, which are then accumulated in non-fatty tissues and cause insulin resistance (2). Normally, obesity is defined by a body mass index (BMI) of ≥30 and is associated with a higher level of the phylum Firmicutes or a higher Firmicutes: Bacteroides ratio (86, 87). Furthermore, the dysbiotic gut of obese people was reported to be high in members of the Prevotellaceae and Enterobacteriaceae families and abundant in the endotoxin-producing genus *Enterobacter* (88). This can lead to gut inflammation and diabetes. Finger millet can be administered to normalize gut dysbiosis as it is a rich source of various indigestible dietary fibers such as arabinoxylan, β-glucan, lignin, cellulose, and resistant starch, that are metabolized by various beneficial gut microbes (Table 4) (9). In particular, non-starchy arabinoxylan fiber in finger millet is determined to be effective in controlling high-fat diet-induced dyslipidemia, hepatic lipid accumulation, and glucose tolerance while enhancing the levels of beneficial gut microbes such as *Lactobacillus*, *Bifidobacterium*, and *Roseburia* (20). Similarly, the β-glucan fiber obtained from finger millet effectively controls the post-prandial glucose levels by inhibiting α-amylase and glucosidase (89). Furthermore, β-glucan decreases cholesterol and triglyceride levels and is also metabolized by gut microbiota to produce SCFAs (90).

**TABLE 4:** Known anti-diabetic and gut-microbiota modulatory effects of different dietary fibers in finger millet.

Fiber	Type	Impact	Model/Study	Reference
β-Glucan	• Water soluble fiber	• ↓ Absorption of glucose in the small intestine • ↓ Firmicutes: Bacteroides • ↑ *Lactobacilli*, ↑ *Bifidobacterium*	• Mice; 6–7 weeks duration • Human; 3 g/day β-glucan for 5 weeks	(9, 90)
Arabinoxylan (hemi-cellulose)	• Water soluble fiber	• ↓ Absorption of glucose in the small intestine • ↓ Hepatic lipid accumulation and inflammation	• HFD mice model • Oral gavage of 0.5 and 1.0 g/kg every alternate day for 10 weeks	(9, 20)
Lignin	• Water insoluble fiber	• ↓ Triglycerides level	• HFD mice model • 0.5% lignophenol in diet	(127)
Cellulose	• Water insoluble fiber	• ↓ Gut Inflammation • ↑ *Alistipes*, ↑ *Akkermansia*	• Germ free mice • 7% cellulose in diet	(128, 129)
Resistant starch	• Water insoluble fiber	• ↑ Insulin sensitivity • ↑ *Bifidobacterium*, ↑ *Eubacterium*	• Mice; 10–30% in diet; 4–10 weeks • Human; 40 g/day for 4 weeks	(130)

As discussed previously, finger millet is rich in polyphenols (3.05–10.20 mg/g GAE) (14, 60) and flavonoids (2–5.54 mg/g CE) (14, 61). Finger millet polyphenols helped maintain lipid metabolism as ferulic acid, the dominant polyphenol in finger millet (35–67 mg/100 g of dry weight) (61) and more prominent in the insoluble fraction of grain (91) resulting in decreased total cholesterol levels (9). In addition, ferulic acid modulated the gut microbiome to improve lipid metabolism and alleviate diabetes (78, 92). In particular, ferulic acid increases the abundance of the mucolytic *Akkermansia* (78), which has anti-obesity and anti-diabetic effects. *Akkermansia* actively participates in glucose homeostasis in the host by releasing Amuc_1100 protein and propionate (2). The Amuc_1100 protein is immunoregulatory and promotes the secretion of serotonin, a glucose-homeostasis neurotransmitter (93). While propionate suppresses blood glucose levels by suppressing gluconeogenesis in the liver increasing the phosphorylation of AMP-activated protein kinase (AMPK) and by enhancing GLP-1 secretion in the gut, which lowers the hepatic visceral fat by reducing triglyceride synthesis (2). Similarly, tannin is also metabolized by the gut microbiota and enhances *Akkermansia* and SCFA-producing microbes of the Lachnospiraceae and Ruminococcaceae families (92). Caffeic acid, another polyphenolic compound in finger millet (14), regulates obesity and fat deposition in the host through gut microbes (94). Administration of caffeic acid restores gut dysbiosis in an obese host and enhances butyrate-producing microbial communities in the gut (94). Similarly, catechin, a flavonoid present in finger millet, is actively metabolized by butyrate-producing *Eubacterium*, probiotic *Bifidobacterium longum*, and *Lactobacillus plantarum*, that improve insulin sensitivity (95), reduces fat accumulation (96), and lowers the blood glucose levels (97), respectively. In addition, finger millet contains phytic acid, a compound of nutraceutical importance, which suppresses hepatic lipogenic gene expression and triglyceride levels, alleviating obesity and associated metabolic disorders (98). In addition, phytic acid also shifts the gut community and enhances the *Bifidobacterium* and *Lactobacillus* (98), which are negatively associated with obesity, and thus their administration can ameliorate obesity.

### 4.2 Microbial regulation of inflammation and oxidative stress

Low-grade or systemic inflammation is associated with insulin resistance and diabetes. This type of inflammation increases the levels of several immunomodulatory molecules, such as IL-1, IL-6, and TNF-α, inducing insulin resistance and regulating C-reactive protein (CRP) (99). CRP is a diabetes marker, and its combination with obesity is positively associated with a higher risk of diabetes (100). Alternatively, the term “oxidative stress” indicates the damaging impact of reactive oxygen species (ROS) and reactive nitrogen species (47) free radicals. Oxidative stress increases glucose overload and advanced glycation products (19, 101). AGEs generate additional ROS and activate the inflammatory cascades, which, along with glucose overload, increases insulin resistance (102).

Gut-dysbiosis also induces systemic inflammation and the risk of diabetes, as a dysbiotic gut permits pathogenic strains and microbial toxins (lipopolysaccharides and enterotoxins) to invade the gut epithelium (2). Bioactive molecules obtained from finger millet are actively metabolized by gut microbes to reduce dysbiosis, and generating metabolites of anti-diabetic significance. For example, catechin, metabolized by the probiotic *L. plantarum*, which is known to reduced low-grade inflammation in diabetes (97). Similarly, gallic acid, another major polyphenol in finger millet, has antioxidant and anti-inflammatory properties (68), which can be metabolized by gut microbes such as *L. plantarum* (103), thereby reducing gut inflammation. Caffeic acid also improves colonic inflammation (colitis) and lowers pro-inflammatory cytokines levels (IL-6, TNF-α, and IFN-γ) through *Akkermansia* (104). *Akkermansia* is a mucolytic microbe enhanced by dietary polyphenol treatment and attenuates obesity-induced metabolic syndromes (105). In addition, arabinoxylan fiber and catechin increases the proportion of *Bifidobacterium* in the host gut and improved high-fat diet-induced diabetes by modulating endotoxemia (106). Endotoxemia is a condition whereby microbial toxins (lipopolysaccharides and enterotoxins) cross the gut epithelial barrier into the bloodstream, causing severe inflammation. *Bifidobacterium* reduces the synthesis of such microbial toxins by regulating gut microbial communities and improving the gut barrier function to prevent the leakage of toxins (106). Furthermore, ferulic acid increases levels of butyrogenic *Faecalibacterium* (78). *Faecalibacterium* is the most abundant butyrate producer and is known for its anti-inflammatory role in the gut, whereby butyrate inhibits NF-κB activation and IL-8 production (107). Furthermore, butyrate produced by *Faecalibacterium* also maintain the integrity of the gut barrier, preventing endotoxemia (108), thereby lowering the risk of diabetes.

### 4.3 Anti-microbial activity to modulate the gut-microbial communities

Selective anti-microbial activity of dietary components maintains a healthy gut environment by limiting the possible pathogens, which lowers the toxin load in the gut, thus with the lower possibility of low-grade inflammation in the gut, which increases insulin resistance and is directly associated with diabetes progression (2, 109). Finger millet modulates microbial communities through the antimicrobial activities of its bioactive molecules, such as tannin and β-glucan. Tannin, a water-soluble polyphenol, reduces the risk of diabetes by inhibiting *Staphylococcus aureus* (110) which causes gastrointestinal infections and releases enterotoxins that compromise gut permeability (111, 112). However, through anti-biofilm activity, β-glucan inhibits the abundance of pathogenic *Enterococcus faecalis*, *Shigella sonnei*, *Proteus vulgaris*, and *Lysinibacillus fusiformis* (89). *E. faecalis* is an opportunistic pathogen associated with several inflammatory diseases that suppresses host immunity (113, 114). Similarly, *S. sonnei* also suppress host immunity in the gut (115, 116), by producing the cytotoxic Shiga toxin-1, causes severe gastrointestinal inflammation (115, 117). *L. fusiformis* and *P. vulgaris* have been reported to cause sepsis, which is a life-threatening hyperreaction to an infection (118) and urinary inflammation (119), respectively. Polyphenolic catechin also inhibits the growth of pathogenic *C. histolyticum* (58). *C. histolyticum* releases cytotoxic toxins, causing apoptosis (120, 121), which could weaken the gut barrier, causing endotoxemia and possibly diabetes.

## 5 Possible anti-diabetic impact of millet induced gut-microbiota

Millets are well known for their health benefits, specially against the obesity and diabetes, and were studied extensively in this regard, but only recently we started recognizing the relevance of gut-microbes in said metabolic conditions. Various millets, except finger millet, are also well-studied for their anti-diabetic role but further studies are still needed to elucidate the exact functional role of gut microbes in this regard. Most of the millets are rich in dietary fiber and polyphenols, yet there is a difference in the level and type of bioactive compound present in an individual millet type (122), which might lead to a slightly different microbial community than others. Although, the association of gut microbes in a millet’s anti-diabetic role is certain, owing to various microbial metabolites produced *via* microbial fermentation of dietary fiber and polyphenols (21). Still, based on the precise data (bio accessibility of and concentration of polyphenols, presence of beta-glucan), this study is focused on the anti-diabetic role of finger millet-induced gut-microbiota. However, other millets are also supposed to have a similar effect with varying degrees of efficacy and microbial markers. Even for finger millet, to date, very few studies have examined the relationship between finger millet and gut microbes, and a lot of things need to be explored regarding millet-induced gut microbiota and their anti-diabetic effect.

## 6 Conclusion

Finger millet is an excellent source of different micronutrients and is rich in complex dietary fibers, which helps to lower the glycemic level and dyslipidemia. Only recently we started recognizing relevance of gut microbes in said metabolic conditions. Therefore, presently much scientific literature is not available to elucidate the multiple aspects of the millet-induced host-microbiome interaction, and a lot of work still needs to be done to understand it. Although, gut-microbes certainly play a critical role in the anti-diabetic effects of finger millet owing to its rich prebiotic content (dietary fiber and polyphenol), which are metabolized by the gut microbiota, to produce various bioactive molecules (SCFAs and phenol) of anti-diabetic significance. In addition, finger millet contains several bioactive molecules that directly affect the beneficial microbial communities in the gut (*Faecalibacterium*, *Lactobacillus*, *Eubacterium*, *Roseburia*, and *Akkermansia*) and thus alleviate systemic inflammation and insulin resistance. However, these bioactive molecules also inhibit various opportunistic pathogens, which limit inflammation and toxin levels in the gut, and further reduce the risk of diabetes in the host. This accumulated evidence suggests that the gut microbiota plays a crucial role in the anti-diabetic effect of finger millet consumption in the host.

## Author contributions

VS conceptualized the manuscript. VS, GL, and MB participated in writing and the illustrations. HS and SA participated in data collection and project management. MB and J-HS supervised, reviewed, and finalized the final draft. All authors contributed to the article and approved the submitted version.

## Footnotes

1
https://www.who.int/health-topics/diabetes#tab=tab_1

2
https://www.nhs.uk/conditions/vitamins-and-minerals
